# Contemporary Review of Subaortic Stenosis Characteristics, Multi-modality Imaging and Management

**DOI:** 10.1007/s11886-025-02287-8

**Published:** 2025-11-27

**Authors:** Aro Daniela Arockiam, Akiva Rosenzveig, Sharmeen Sorathia, Rishabh Khurana, Ankit Agrawal, Elio Haroun, Rochell Issa, Anoop Gurram, Mohammad Alamer, Tiffany Dong, Joseph El Roumi, Muhammad Majid, Leonardo Rodriguez, Zoran B. Popovic, Brian P. Griffin, Tom Kai Ming Wang

**Affiliations:** Section of Cardiovascular Imaging, Department of Cardiovascular Medicine, Sydell and Arnold Miller Heart, Vascular, and Thoracic Institute, Cleveland Clinic, 9500 Euclid Avenue, Main Campus, J1-5, Cleveland, OH 44195 USA

**Keywords:** Subaortic stenosis, Congenital heart disease, Echocardiography, Cardiac computed tomography, Cardiac magnetic resonance, Cardiac surgery

## Abstract

**Purpose of Review:**

This review aims to provide a comprehensive contemporary overview regarding the clinical perspectives, multi-modality imaging evaluation, treatments and outcomes of subaortic stenosis (SAS).

**Recent Findings:**

SAS remains an important condition making up a significant minority of patients with progressive fixed left ventricular ouflow tract obstruction. Echocardiography remains the first-line imaging modality to diagnose SAS, evaluate severity of obstruction along with cardiac chamber and valvular function. Transesophageal echocardiography, cardiac computed tomography and cardiac magnetic resonance have adjunctive roles to help delineate SAS anatomy, functional implications and pre-operative planning. A variety of surgical techniques have been developed for SAS with significant obstruction often with symptoms, with excellent contemporary outcomes, though recurrence rates remain significant particularly in younger patients and those with complex anatomical features that may need repeat surgeries.

**Summary:**

Multi-disciplinary approach to management is necessary to improve clinical outcomes, including multi-modality imaging for diagnosis, risk stratification, treatment guidance and close surveillance, along with meticulous surgery by experienced surgeons, are necessary to improve clinical outcomes for SAS.

**Graphical Abstract:**

Pathophysiology, Evaluation, and Management of Subaortic Stenosis.

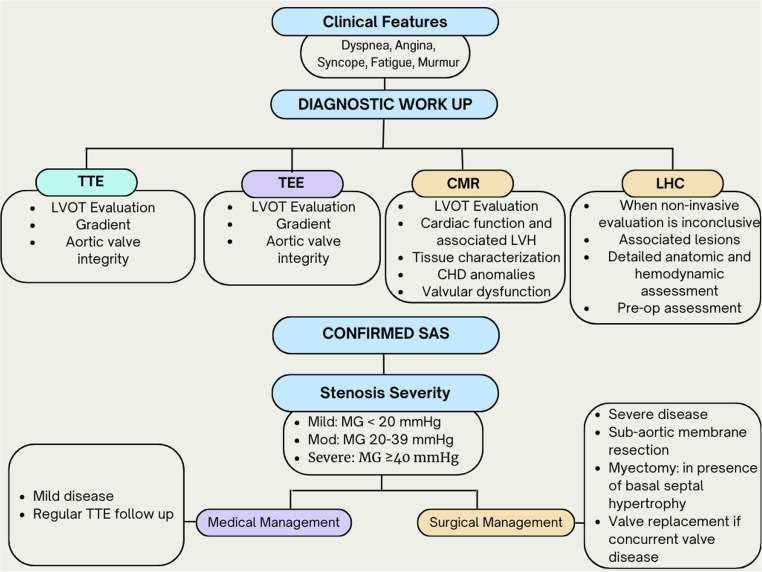

## Introduction

Sub valvular aortic stenosis, also known as subaortic stenosis (SAS), is a rare heart defect characterized by the presence of a discrete fibro-muscular membrane beneath the aortic valve that can lead to left ventricular outflow tract (LVOT) obstruction. Although SAS is classified as a congenital anomaly, it is not the result of a primary genetic disorder, and it usually develops within the first decade of life secondary to morphological abnormalities in the LVOT [1–4]. SAS typically follows a progressive clinical course where more than 80% of untreated patients experience progressively worsening obstruction and aortic regurgitation (AR) [5] Transthoracic echocardiography (TTE) remains the first-line diagnostic imaging modality, however transesophageal echocardiography (TEE), computed tomography (CT), cardiac magnetic resonance (CMR), and cardiac catheterization have important complimentary roles in assessment of SAS [5]. When indicated, surgical resection by way of a transaortic approach is the preferred interventional treatment; however, recurrence rates are high [4]. This review provides a comprehensive overview of the contemporary perspectives of the diagnosis, management and outcomes in individuals with SAS.

## Anatomy and Pathophysiology

SAS includes various types of abnormalities that may appear as an isolated entity or in combination with other structural defects. Commonly observed defects include a slim membrane (which is the most frequent type), a dense fibromuscular ridge [5–7], widespread tunnel-shaped obstructiis a circumferential protruding fibroon, irregular attachments of the mitral valve, and, rarely, additional endocardial cushion tissue. SAS can result in a fixed or a dynamic form of LVOT obstruction, the latter typically being associated with hypertrophic cardiomyopathy (previously termed idiopathic hypertrophic subaortic stenosis) [6]. SAS leading to a fixed obstruction manifests in two distinct phenotypes The discrete type, occurring in 70–90% of cases [6, 7], is a circumferential protruding fibrous membrane below the aortic valve as shown in graphical abstract. The less common type is that of a fibromuscular tunnel-like diffuse narrowing within the outflow tract. This membrane’s position varies, starting below the aortic valve and extending deeper into the left ventricle. In certain instances, the subaortic tissue may progressively restrict the base of the aortic valve leaflets, contributing to LVOT obstruction and/or AR. Moreover, up to three-quarters of affected patients exhibit some form of left ventricular septal hypertrophy due to increased afterload [7]. Damage to the aortic leaflets by high-velocity jets induced by the subaortic stenosis leads to AR in 30–80% of patients [4, 7, 8]. Furthermore, the decrease in aortic diastolic pressure decreases coronary perfusion, thus increasing left ventricular oxygen demand and the risk of myocardial ischemic injury [7, 8]. 

Fixed SAS arises from a complex interplay of factors, including genetic predispositions, hemodynamic changes seen in other cardiac anomalies, and specific anatomical features of the LVOT that exacerbate flow disturbances. Conditions like a constricted LVOT, significant aortic override, and a steep atrioventricular septal angle, for example, are known to disrupt flow consistently. In a pivotal 1979 study, Rosenquist et al. [9] examined 22 heart specimens affected by SAS and noted that the space between the mitral and aortic valves in patients with SAS was significantly larger compared to healthy hearts. This finding led to the hypothesis that a greater mitral-aortic gap, altering the angle of blood ejection from the left ventricle during crucial developmental stages, might be a key predisposing factor in SAS development. Such alterations could stimulate embryonic cells near the ventricular septum to coalesce into a fibroelastic ridge or band. Supporting this idea, research by Sigfússon et al. and subsequent fluid modeling studies have demonstrated that a more acute aorto-septal angle alters shear forces, potentially leading to SAS [10]. These changes in fluid dynamics are known to drive endothelial and muscular cell proliferation, eventually forming a fibromuscular ridge. This mechanistic pathway is thought to play a central role in the development of SAS [11, 12]. Moreover, the prevalence of SAS in patients with other congenital heart defects, reported to be as high as 6.5%, suggests a relationship with altered cardiac anatomy [12]. Surgical interventions to correct other congenital defects can potentially modify the left-sided outflow, further increasing turbulence and shear stress on the interventricular septum, thereby contributing to the formation of SAS. This cumulative effect of genetic, developmental, and hemodynamic factors underscores the complexity of SAS pathogenesis [13, 14].

### Epidemiology and Associated Conditions

Sub valvular aortic stenosis (SAS) represents 8–20% of etiologies of LVOT obstruction [6]. SAS has historically been described in children and most commonly diagnosed before the age of 10. However, more recent data has described SAS in adults as well, with one single center study noting a prevalence of SAS in 6.5% of adult patients with congenital heart disease [15]. Sung et al. noted that adults comprised 26% of all surgeries performed for SAS correction [16]. SAS affects males predominantly, constituting 60% of all SAS cases [7]. About 50–70% of cases overall and 44% of adult cases are associated with other congenital heart defects [7, 16]. SAS is most commonly associated with ventricular septal defects (23–37%), but has also been associated with patent ductus arteriosus (34%), bicuspid aortic valve (22.7%), interrupted aorta including coarctation (23%), and others [7]. Although rare, SAS has also been described as part of a syndrome in the case of Shone’s syndrome, Noonan’s syndrome, and rubella syndrome [17–19].

### Clinical Manifestations

The clinical presentation of SAS varies widely based on the severity of the obstruction. In mild cases, it may be asymptomatic and is only discovered incidentally with a systolic ejection murmur loudest at the left-mid sternal border radiating to the left-upper sternal border during routine examination, or during imaging studies. Moreover, a diastolic murmur may signify concomitant AR. However, as the obstruction worsens, patients are more likely to exhibit symptoms related to impaired cardiac function and increased pressure within the left ventricle. Symptoms of SAS include exertional dyspnea, decreased exercise tolerance, angina, presyncope, syncope, and sudden cardiac death. Exertional dyspnea is the most common manifestation, occurring in 40% of symptomatic cases. It occurs due to increased left ventricular filling pressures caused by left ventricular hypertrophy (LVH) with reduced diastolic compliance [20].

### Electrocardiography

Electrocardiography findings meeting voltage criteria for LVH and findings of left heart strain such as ST depressions and T wave inversions in the left-sided leads is a common finding in SAS but are neither sensitive nor specific for the diagnosis of this condition. These findings, however, should prompt the physician to pursue further investigations for left ventricular structural abnormalities by the means of cardiac imaging modalities such as transthoracic echocardiography [13].

### Echocardiography

**Fig. 1:**
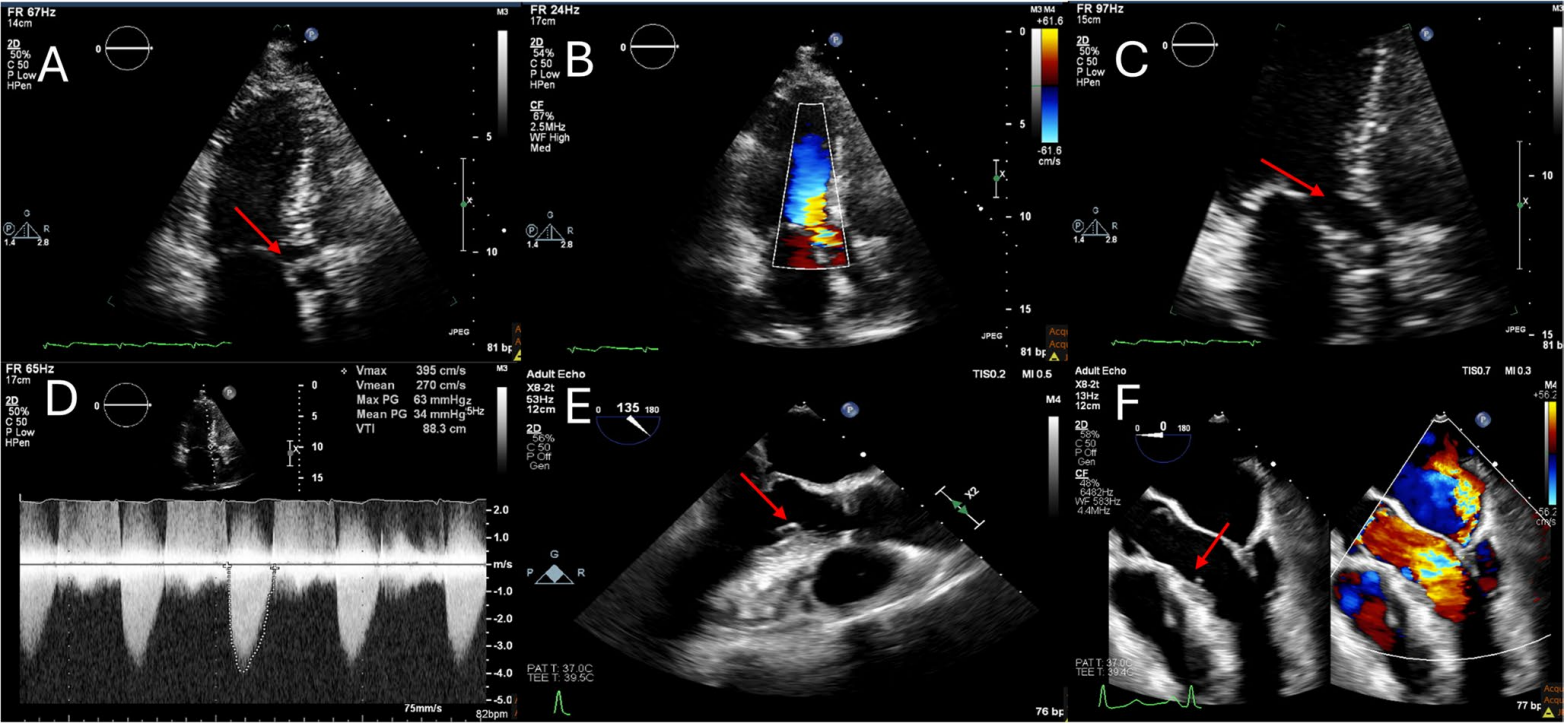
(**A**) Subaortic membrane (arrow) visualized on 2D echocardiography. (**B**) Flow acceleration at the level of the membrane (**C**) Subaortic membrane (arrow) visualized on 2D echocardiography with continuous wave Doppler showing a fixed obstructive profile with peak velocity of 4.0 cm/s (**D**) mean gradient of 34mmHg (**E**) Transesophageal echocardiography showing a subaortic membrane (arrow) on 2D (**F**) Another patient transesophageal echocardiography showing subaortic membrane (arrow) with flow acceleration by color Doppler at the level of the membrane along with significant mitral regurgitation

Echocardiography is the first-line imaging modality for evaluating the anatomical and physiological features of SAS. The classification of SAS based on echocardiographic findings is primarily determined by the pressure gradients and peak velocities across the LVOT. According to the 2018 American Heart Association/American College of Cardiology (AHA/ACC) Guidelines for the Management of Adults with Congenital Heart Disease, severity is divided into mild, moderate, and severe SAS [1, 5]. Mild SAS is defined as a mean gradient of less than 20 mmHg and a peak velocity of less than 3.0 m/s. Moderate SAS is defined as a mean gradient of 20–39 mmHg, and a peak velocity of 3.0–3.9 m/s [5]. Lastly, severe SAS is defined as a mean gradient of greater than 40 mmHg, and a peak velocity of greater than 4.0 m/s [5]. These classifications are based on Doppler echocardiographic measurements, which are essential for assessing the hemodynamic impact of the obstruction. When TTE has suboptimal windows or inconclusive findings, TEE may add additional information such as allow higher resolution of the site and extent of the membrane as shown in Fig. 1.

Longer-term surveillance imaging for SAS is important. In infants who have not been operated on, surveillance echocardiography should be completed every 1–3 months for any degree of SAS with *≤* mild AR. For children and adults that have not been operated on, routine surveillance should be completed every 1–2 years for mild SAS without AR [11]. For asymptomatic patients with stable LVOT obstruction and mean gradients of < 30 mmHg but without LVH and significant AR, annual echocardiography is recommended for routine surveillance to monitor for increasing obstruction, development or progression of AR, and evaluation of systolic and diastolic left ventricular function as shown in graphical abstract [5, 9, 12]. Meanwhile, for moderate to severe SAS, TTE is appropriate for general surveillance for all indications, alongside consideration of TEE and CMR [11]. For postoperative patients, TTE is appropriate for general surveillance for all indications [11]. Postoperative asymptomatic patients should undergo annual echocardiography to assess for recurrent obstruction and AR, which can occur despite surgical intervention. This is also recommended to evaluate for progressive or recurrent obstruction and AR, which is thought to occur at a rate of about 20% over 10 years post resection [9, 12]. Overall, the ACC and AHA guidelines emphasize the importance of lifelong cardiology follow-up, including evaluation by or consultation with an adult congenital heart specialist for patients with SAS [1].

Stress echocardiography is a valuable tool in the evaluation of SAS, particularly for the assessment of the dynamic nature of the obstruction and its hemodynamic impact under stress conditions. The 2018 AHA/ACC Guideline for the Management of Adults with Congenital Heart Disease provides specific recommendations for the use of stress testing in this context. Exercise stress echocardiography (ESE) is also recommended for the evaluation of exercise capacity, symptoms, electrocardiographic changes, and arrhythmias in patients with SAS, especially when there are equivocal indications for intervention [1, 5]. For ESE, upright exercise, such as treadmill or bicycle, is preferred as these methods reflect the more physiologic form of exercise and provokes higher gradients compared to supine exercise. The Valsalva maneuver, particularly the strain phase, is used during stress echocardiography as it increases intrathoracic pressure and decreases venous return, which can precipitate LVOT obstruction [10]. However, the limitation of the Valsalva maneuver is that the response can be variable due to the subjective nature of the effort. Lastly, amyl nitrite can be used to assess SAS, as it decreases afterload which increases the gradient across the LVOT. Amyl nitrite provokes significant gradients and is often used in conjunction with other stress modalities. In summary, stress echocardiography and provocative maneuvers can be useful in the evaluation of SAS to unmask LVOT gradients and to assess the dynamic nature of the obstruction.

### Cardiac Magnetic Resonance

**Fig. 2:**
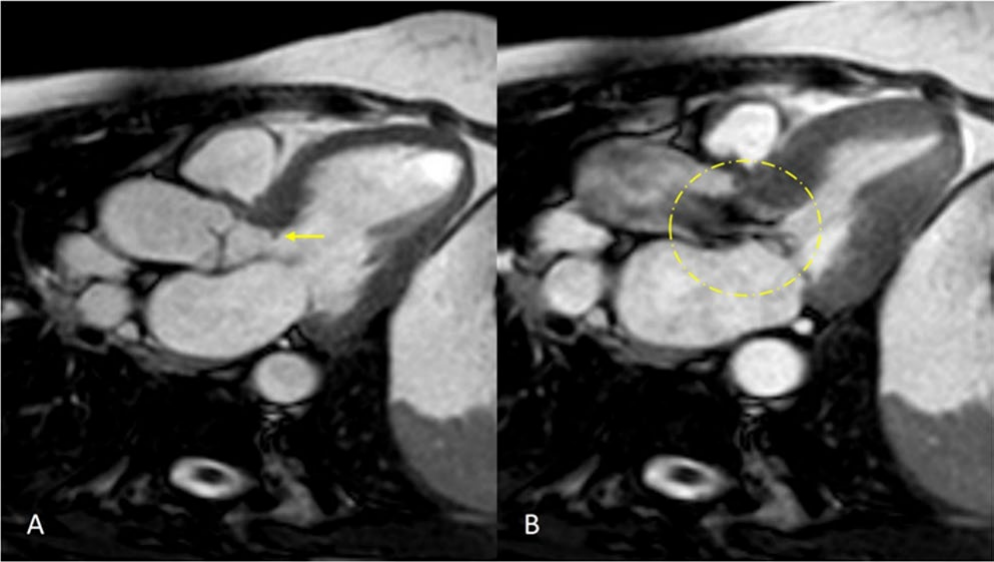
Cardiac Magnetic Resonance Imaging, three chamber view showing subaortic membrane (**A**, yellow arrow) with dephasing jet across left ventricular outflow during left ventricular systole (**B**)

Cardiac magnetic resonance (CMR) imaging offers unique advantages in the assessment of subaortic stenosis. It provides comprehensive assessment of ventricular volumes, ejection fraction, and myocardial mass, which are essential parameters for evaluating cardiac function and identifying associated ventricular hypertrophy secondary to subaortic obstruction. Additionally, CMR can assess myocardial tissue characteristics using techniques such as T1 and T2 mapping, late gadolinium enhancement (LGE) which can help in detecting myocardial fibrosis, inflammation, or ischemia. Furthermore, CMR offers superior soft tissue contrast compared to CT, which allows better visualization of the aortic valve leaflets, sub-valvular apparatus, and surrounding myocardial structures. It is pertinent to mention that the region of interest may be hidden by the spin dephasing artefact making visualization of thin subaortic membranes challenging. Using flow analysis, dynamic assessment of blood flow through the stenotic segment can be performed, enabling accurate quantification of pressure gradients and flow velocities across the stenotic lesion (Fig. 2). This information is crucial for determining the severity of subaortic stenosis and guiding treatment decisions. Moreover, lack of ionizing radiation makes CMR a safe imaging modality for serial follow-up evaluations in paediatric patients, who may require long-term monitoring for disease progression or response to therapy. Additionally, CMR can provide valuable information for concomitant congenital heart disease anomalies, cardiac chamber size and function quantification, valvular dysfunction and aortic pathology, some of which are known to co-exist with SAS [21]. However, CMR has not replaced echocardiography as the standard of care due to it increased cost.

### Computed Tomography

**Fig. 3:**
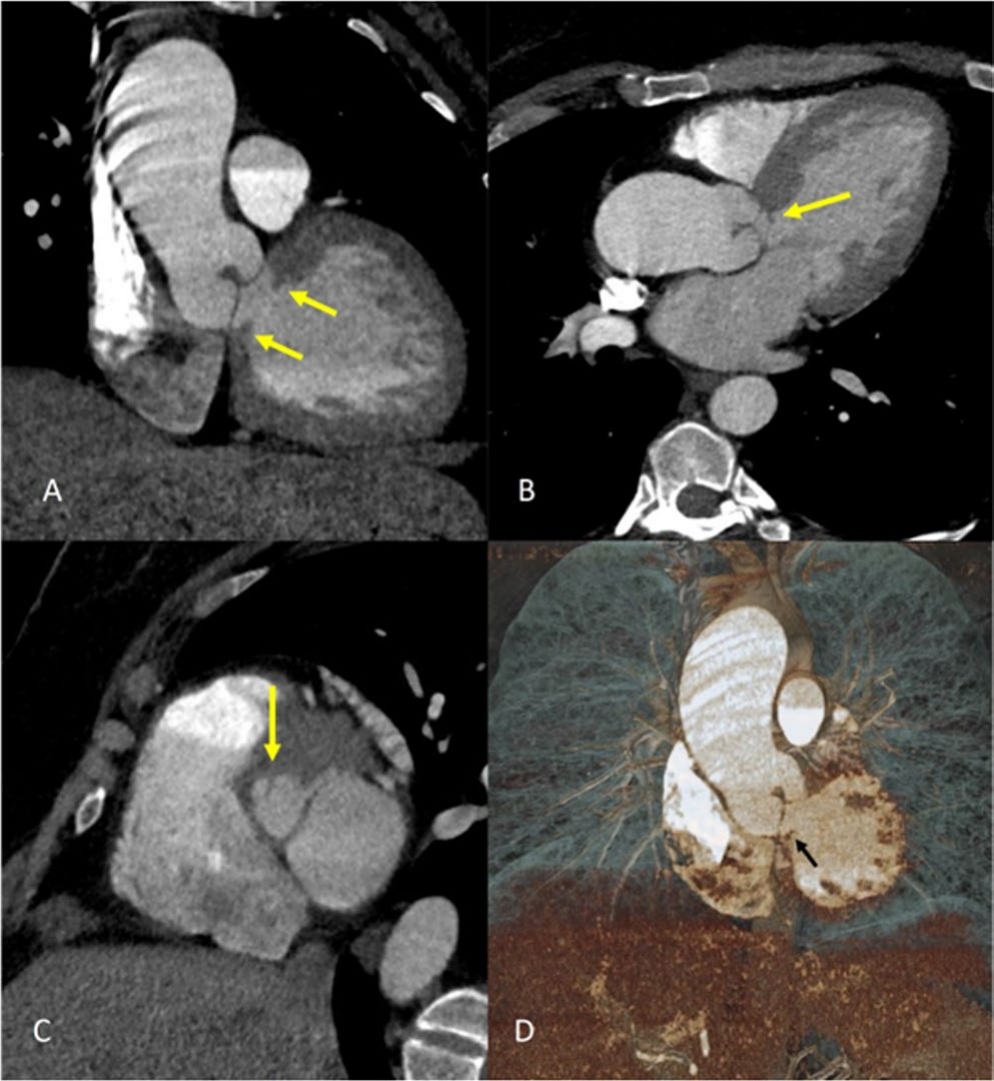
Computed Tomography Angiography Multiplanar Reconstruction Images (**A**-**C**) showing linear hypodensity (yellow arrows) in the left ventricular outflow suggesting subaortic membrane. **D**) Cinematic Volume Rendered Computed Tomography image showing subaortic membrane (black arrow)

When CMR is contraindicated or not available, cardiac computed tomography angiography (CTA) may be performed since it offers high spatial resolution and rapid acquisition times, with minimal patient discomfort, making it particularly useful for assessing the anatomy of the heart and great vessels in patients with subaortic stenosis. Using CTA, a detailed three-dimensional reconstruction of the aortic root, LVOT, and surrounding structures can be generated, thereby allowing for precise localization and characterization of the stenotic lesion (Fig. 3) Furthermore, CT can accurately measure the dimensions of the stenotic segment, including its length, diameter, and degree of narrowing, assess calcifications, which are essential parameters for determining the severity of the obstruction and aid in planning surgical or interventional procedures. Retrospective 4D CTA of the aortic root can enable the generation of cine images to enable better anatomical delineation. Additionally, CT imaging can provide valuable insights into the presence of associated cardiac and coronary anomalies [22, 23].

### Cardiac Catheterization

Cardiac catheterization in SAS is generally reserved for specific clinical scenarios where noninvasive imaging is insufficient or when additional hemodynamic information is required. According to the 2018 AHA/ACC Guideline for the Management of Adults with Congenital Heart Disease, the indications for cardiac catheterization in SAS include inconclusive noninvasive imaging, associated lesions, preoperative assessment, and evaluation of symptoms. When echocardiography or other noninvasive imaging modalities provide conflicting or inconclusive data regarding the severity of the obstruction or associated lesions, cardiac catheterization can be used to obtain accurate hemodynamic measurements. Additionally, cardiac catheterization is indicated when SAS is associated with other congenital or acquired cardiac lesions that require detailed anatomical and hemodynamic assessment [1, 12]. In the case of patients being considered for surgical intervention, cardiac catheterization may be performed to precisely measure the sub valvular gradient and to evaluate coronary anatomy, especially if there is a suspicion of coronary artery involvement or other complex anatomical considerations [1]. Lastly, cardiac catheterization may be indicated in symptomatic patients in which the severity of SAS does not correlate with the clinical presentation to help in the assessment of true hemodynamic impact of the obstruction [2]. Table 1 provides an overview of the strengths and limitations of the various imaging modalities.

**Table 1:** Key features, strengths and limitations of multimodality imaging for evaluating SAS

Imaging modality	Key features	Strengths	Limitations
Transthoracic echocardiography	Visualizing the subaortic region and assess the morphology and severity of the stenosis using two-dimensional imaging.	♣ Non-invasive ♣ Assessment of associated anomalies, for example septic or valvular (regurgitation). ♣ Easier to perform, including basic use of parasternal long axis and apical 5 and 3 chamber views. ♣ Doppler provides critical values of peak and mean pressure gradients guiding therapy.	♣ Quality of images can be limited due to poor acoustic windows
Transesophageal echocardiography	Three-dimensional visualization	♣ Superior image quality than transthoracic leading to more accurate parameters ♣ Higher utility when TTE has limited acoustic windows	♣ Invasive
Computed tomography angiography	High spatial resolution and rapid acquisition times with 3D reconstruction	• 3D allows for precise visualization • Accurate measurements of dimensions • Retrospective 4D for better anatomic delineation • Additional information on associated anomalies	• Noninvasive • Minimal patient discomfort • Ionizing radiation
Cardiac Magnetic Resonance Imaging	Assessment of ventricular volumes, ejection fraction myocardial mass and tissue characteristics	• Additional information with late gadolinium enhancement to detect fibrosis, inflammation or ischemia • Soft tissue contrast superior to CT • Dynamic assessment of blood flow • Safe modality without use of ionizing radiation • Long term monitoring in pediatric patients • Can provide information about associated anomalies	• Non-invasive • Artefacts can make thin subaortic membranes difficult to visualize
Cardiac catheterization	Direct measurement of hemodynamic data to assess pressure gradients across the stenosis	♣ Provides direct measurements of intracardiac pressures to assess pressure gradients across stenotic valve. ♣ Intracardiac echocardiography gives real-time imaging of subaortic region. ♣ FFR ♣ Additional information about morphology of the stenosis with angiography ♣ Selective angiography of LVOT to aid in approach of intervention. ♣ Needs more definitive information with noninvasive modalities have uncertain or discordant information	♣ Invasive

*TTE* Transthoracic echocardiography, *CT* Computed tomography, *LVOT* Left ventricle outflow tract obstruction, *FFR* Fractional flow reserve

### Pharmacological Management

There is currently no role for medical therapy for management of patients with subaortic stenosis and there are no medications approved for this indication. Close follow-up of asymptomatic patients who do not meet criteria for surgical intervention is a reasonable approach. If patients develop symptomatic heart failure or left ventricular dysfunction, they are treated with heart failure guideline-directed medical therapy until surgery can be performed.

### Surgical Intervention

SAS poses a unique challenge to affected patients and can cause clinical sequelae of LVOT obstruction. In certain situations, patients can benefit from surgical relief of the obstruction. Short-term surgical outcomes for SAS are typically satisfactory, yet recurrence rates are relatively high, ranging from 0 to 55% [7, 20], many of whom (ranging from 0 to 31%) [7, 21] requiring reintervention. The surgical approach for SAS correction varies depending on the type of SAS present. For instance, discrete SAS surgery includes a membranectomy and a myotomy or myectomy if septal hypertrophy is present. For tunneled SAS, other surgical approaches include myotomy, extensive myectomy, apical conduit insertion, the Konno-Rastan procedure, and the modified Konno procedure [6, 24, 25]. The anterior aortoventriculoplasty, also known as the Konno-Rastan procedure, involves opening the right ventricular outflow tract (RVOT) and cutting through the aortic annulus and the ventriculo infundibular fold into the ventricular septum [13, 26]. The Konno-Rastan approach has demonstrated good results with a 24-year single center study of 53 patients showing 86% 10-year survival and 11% incidence of complete heart block (CHB) [27]. The Modified Konno, or subaortic ventriculoplasty, is a similar procedure, but spares the aortic valve [28]. Surgical correction carries its risks as well, including general surgical complications and a 10–15% risk of CHB [1]. The choice of surgical technique should carefully balance the benefits of reducing the risk of reobstruction against the increased risk of CHB and the consequent need for pacemaker implantation. The 2018 AHA/ACC guidelines for the management of adults with congenital heart disease and the 2020 European Society of Cardiology (ESC) guidelines for the management of adult congenital heart disease provide recommendations for the management of SAS as an isolated lesion, or in association with AR (Table 2); however, no recommendations are provided for more complex lesions. The absence of specific recommendations for complex lesions stems from a paucity of robust data and intricate anatomic variations, urging caution in extrapolation [1, 28]. The 2018 AHA/ACC guidelines give a class I indication for intervention of SAS when peak gradient is 50 mmHg or more and the patient has symptoms attributable to SAS or if the gradient is less than 50 mmHg but the patient has heart failure or ischemic symptoms. However, a class IIb recommendation is given for asymptomatic patients who have mild or worse AR and a max gradient of 50 mm Hg [1]. The ESC guidelines differ with their recommendations. They give a Class I recommendation for those who are symptomatic with a mean gradient of 40 mmHg or more or severe AR. However, they give a Class IIa recommendation for intervention for asymptomatic patients who have ejection fraction less than 50%, marked left ventricular hypertrophy, or fall in blood pressure during exercise. Importantly, ESC guidelines also give a Class IIb recommendation for asymptomatic patients with a mean gradient of 40 mmHg or higher or if there is concern for progression of AR (Table 2). Post-operative follow-up is necessary to monitor for complications such as arrhythmias, CHB and iatrogenic ventricular septal defect as well as to monitor for recurrence and progressive AR.

**Table 2:** Guidelines indications for surgery intervention

Guideline	Recommendation	Patient Characteristics	Level of Evidence
AHA/ACC 2018	Class I	Maximum gradient ≥ 50 mmHg and symptomatic	C
AHA/ACC 2018	Class I	Maximum gradient < 50 mmHg and heart failure/ischemic symptoms or LV systolic dysfunction	C
ESC 2020	Class I	Symptomatic patients with mean Doppler gradient ≥ 40 mmHg or AR	C
AHA/ACC 2018	Class IIb	Asymptomatic adults with subAS, at least mild AR, and maximum gradient ≥ 50 mmHg	C
ESC 2020	Class IIa	Asymptomatic patients with mean gradient < 40 mmHg but LVEF < 50%	C
ESC 2020	Class IIa	Asymptomatic patients with AR, LVESD > 50 mm (or 25 mm/m2 BSA), and/or EF < 50%	C
ESC 2020	Class IIa	Asymptomatic patients with mean Doppler gradient ≥ 40 mmHg and marked LVH	C
ESC 2020	Class IIa	Asymptomatic patients with mean Doppler gradient ≥ 40 mmHg and fall in blood pressure below baseline on exercise	C
ESC 2020	Class IIb	Asymptomatic patients with mean Doppler gradient ≥ 40 mmHg, normal LV (EF > 50%, no LVH), normal exercise testing, and low surgical risk	C
ESC 2020	Class IIb	Asymptomatic patients with documented progression of AR beyond mild	C

*AHA/ACC* American Heart Association/American College of Cardiology, *ESC* European Society of Cardiology, *LV* Left ventricle, *AR* aortic regurgitation, *LVEF* Left ventricle ejection fraction, *LVESD* Left ventricle end-systolic diameter, *LVH* Left ventricle hypertrophy, *EF* Ejection fraction, *BSA* Body surface area

### Prognosis and Complications

Surgical resection of the subaortic membrane in children typically has a low rate of mortality. Despite this, recurrence and reoperation rates are high. AR is common following SAS resection in pediatric patients, though its severity usually remains mild. However, it can progress over time in the case of concomitant congenital aortic stenosis (AS), necessitating close follow up into adulthood. Moreover, Preoperative peak LVOT gradient > 80 mmHg predicted the progression to moderate AR in the postoperative period. The rates of SAS recurrence post-surgery vary widely in the literature, with reported recurrence rates ranging from 15 to 40%. This variability is influenced by several factors including the surgical technique employed, the extent of resection, and the underlying pathology of stenosis. For example, a study by van der Linde et al. noted a recurrence rate of approximately 25% within a decade post-surgery [29]. According to this study, patients who underwent surgery before the age of 5 had a recurrence rate of approximately 40%, compared to 20% in older children and adults [29] (Table 3). In a large multicenter study of adult patients who underwent surgical resection for SAS, 26% required reoperation with a median duration of follow up of 12.9 years [8, 29]. Reoperation is common especially in cases with higher preoperative LVOT gradient and in female patients. Predictors of need for reoperation include distance less than 6 mm between the aortic valve and the obstruction, a peak gradient by doppler ≥ 60mmHg, younger age at resection, peeling of the membrane of the aortic valve or mitral valve, and presence of AS. Additional myectomy did not improve outcomes and increased the risk of CHB requiring pacemaker [21, 29].

**Table 3:** Key studies of SAS (with > 100 patients) and their management and outcomes

Study		Year	Country	Number of patients	Median/mean age, gender	Surgery	Outcomes
Bandara et al.		2023	Australia	120	• 4.7 years • 59% male	Fibrous tissue excision with septal myectomy	• Median follow up of 13 years • Recurrence in 18% (20), with 15 undergoing reinterventions • Freedom from intervention: 99%, 94%, 93% and 90% at 1, 3, 5 and 10 years
Schlein et al.		2022	Austria	103	• 5.5 years • 56.3% male	Transaortic septal myectomy, with resection of redundant sub valvular membrane tissue	• 30 years follow up • 90.8% survival at 10 years and 88.7% at 20 and 30 years. • Reoperation in 21.6% at 10 years, 28.2% at 30 and 30 years
Pickard et al.		2015	United States of America	155	• 5.2 years at the time of surgery • 60.6% male	Discrete subaortic stenosis resection with additional myectomy in 50% patients	• Median follow up of 10.9 years • 21% had reoperation, plateaued after 10 years • 98.6% survival at 10 years and 86.3% at 20 years
Dorobantu et al.		2014	United Kingdom	1047	• 6.5 years • 61.5% male	Konno type operation	• 12 years follow up • Overall, 98% 30-day survival, 92% freedom from reintervention at 5 years and 88.5% at12 years • Simple stenosis had better outcomes than complex stenosis
Linde et al.		2013	Multicenter (Netherlands, Canada, Belgium)	313	• 17.1 at the time of first surgery • 52% male	Isolated enucleation with/without additional myectomy	• Median follow up 12.9 years • 25.6% required at least 1 reoperation. • Mean time interval to first reoperation: 12 years. • Median intervention free survival: 17 years.
Barboza et al.		2012	Guatemala	113	• 7 years • 54% female	For isolated subaortic membrane: Subaortic membrane resection + myectomy in 20%	• Mean follow up of 3.4 years. 3 groups of isolated subaortic membrane, subaortic membrane + ventricular septal defect, and SM + patent ductus arteriosus. • 15% had residual subaortic membrane. 27% of those with no postop residual subaortic membrane had recurrence, with mean time to develop subaortic membrane being 2 years.
Geva et al.		2007	United States of America	112	• 5.1 years at the time of first surgery • 61% male	Endarterectomy type of resection of fibroelastic tissue + 52% myectomy	• Median follow up 8.2 years • 14% required reoperation, with median time 6.9 years
Ruzmetov et al.		2005	United States of America	190	• 8.2 years • 60.5% male	Subaortic membrane resection with myectomy in 14.6%	• 4% early and 5% late deaths • Follow up of 7.1 years • 28% had reoperations
Van Son et al.		1993	United States of America	169	• 15.3 years • 51.5% female	Membranectomy alone in 33.8%, with myotomy in 15.6%, and with myectomy in 46.1%	• 4.7% early and 9.4% late deaths • Reoperation in 15.3% • Risk of late aortic insufficiency least with myectomy
Vogt et al.	1989		Germany	132 operated	• 7.8 years • 64% male	93% had conventional surgical techniques	• 8% early and 2% late deaths • Reoperation in 15% • 33% of operations led to satisfactory hemodynamic results
Agrawal et al.	2024		United States of America	484	• 5.5 years• 67.5% female	48.6% had surgery	• Median follow up of 5.5 years• 11.5% Mortality• 6.8% Heart Failure Hospitalization

The gradual increase in the LVOT gradient persists over time. For most patients, follow-up intervals of 2 to 4 years suffice due to the slow progressive nature of the obstruction. Ultimately, most patients will necessitate reoperation for recurring SAS at some point in their lives. Reoperation due to recurring discrete subaortic stenosis is a frequent occurrence, with reported rates ranging between 6% and 30% [8, 29]. Studies addressing the risk of reoperation following relief of subaortic obstruction have primarily focused on various anatomic subtypes [21, 29]. Notably, two specific high-risk subgroups for recurrence and subsequent reoperation have been clearly delineated: patients with a tunneled SAS and those exhibiting multilevel LVOT obstruction [24, 29]. Recommendations suggest that individuals displaying a residual left ventriculo-aortic gradient exceeding 30 mmHg intra-operatively should undergo more aggressive subaortic resection [24].

## Conclusion

This review summarized the characteristic clinical, multi-modality imaging, interventional and prognostic features of SAS. This entity constitutes 6.5% of adult congenital heart conditions, leading mainly to a fixed outflow tract obstruction. Echocardiography is the workhorse imaging tool for diagnosing and grading severity of SAS, whilst other cardiac imaging modalities have complimentary roles. The preferred treatment involves surgical correction, generally yielding an excellent prognosis, although regular surveillance is necessary post-operatively due to significant recurrence rates, especially in predisposed patients.

## Key References

- Stout KK, Daniels CJ, Aboulhosn JA, Bozkurt B, Broberg CS, Colman JM, et al. 2018 AHA/ACC Guideline for the Management of Adults with Congenital Heart Disease: A Report of the American College of Cardiology/American Heart Association Task Force on Clinical Practice Guidelines. Circulation [Internet]. 2019 Apr 2 [cited 2024 Apr 22];139(14).

- This reference reports the ACC/AHA guidelines for evaluation and management of adults with congenital heart disease.

- Contemporary Clinical Characteristics, Imaging, Management, and Surgical and Nonsurgical Outcomes of Adult Patients With Subaortic Stenosis. J Am Heart Assoc 2024;13:e036994.

- This reference reports a large contemporary cohort study of subaortic stenosis patients including clinical characteristics, imaging, management and outcomes.

## Data Availability

No datasets were generated or analysed during the current study.
